# 3,12-Diaza-6,9-diazo­nia-2,13-dioxotetra­decane bis­(perchlorate)

**DOI:** 10.1107/S1600536811055516

**Published:** 2012-01-11

**Authors:** Tilo Söhnel, Kathrin A. Wichmann, Thomas Doert, Garth J. S. Cooper

**Affiliations:** a School of Chemical Sciences, The University of Auckland, Private Bag 92019, Auckland, New Zealand; b School of Biological Sciences, The University of Auckland, Private Bag 92019, Auckland, New Zealand; c Department of Chemistry and Food Chemistry, Technical University of Dresden, 01062 Dresden, Germany; d Centre for Advanced Discovery and Experimental Therapeutics, NIHR Manchester Biomedical Research Centre, Central Manchester University, Hospitals NHS, Foundation Trust, York Place, Manchester M13 9WL, England; e School of Medicine, University of Manchester, Oxford Road, Manchester M13, England

## Abstract

The crystal structure of the title diprotonated diacetyl­triethyl­ene­tetra­mine (DAT) perchorate salt, C_10_H_24_N_4_O_2_^2+^·2ClO_4_^−^, can be described as a three-dimensional assembly of alternating layers consisting of diprotonated diacetyl­triethyl­ene­tetra­mine (H_2_DAT)^2+^ strands along [100] and the anionic species ClO_4_^−^. The (H_2_DAT)^2+^ cations in the strands are connected *via* N—H⋯O hydrogen bonding between the acetyl groups and the amine groups of neighbouring (H_2_DAT)^2+^ cations. Layers of (H_2_DAT)^2+^ strands and perchlorate anions are connected by a network of hydrogen bonds between the NH and NH_2_ groups and the O atoms of the perchlorate anion. The asymmetric unit consits of one perchlorate anion in a general position, as well as of one cation that is located on a center of inversion.

## Related literature

For background to pharmaceutical chelating agents in the treatment of diabetes, see: Cooper *et al.* (2004 ▶); Gong *et al.* (2006 ▶, 2008 ▶); Jüllig *et al.* (2007 ▶); Lu *et al.* (2010 ▶). For the detection of a new group of TETA metabolites, see: Lu *et al.* (2007 ▶). For the preparation and characterization of DAT mono- and dihydro­chloride salts, see: Jonas *et al.* (2006 ▶); Wichmann *et al.* (2011 ▶). For related structures, see: Elaoud *et al.* (1999 ▶); Fu *et al.* (2005 ▶); Ilioudis *et al.* (2000 ▶, 2002 ▶); Ilioudis & Steed (2003 ▶); Wichmann *et al.* (2007 ▶). 
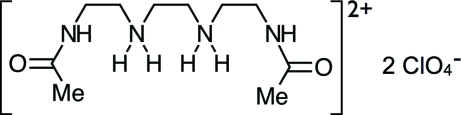


## Experimental

### 

#### Crystal data

C_10_H_24_N_4_O_2_^2+^·2ClO_4_^−^
*M* *_r_* = 431.23
Monoclinic, 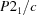

*a* = 6.0888 (5) Å
*b* = 10.9415 (9) Å
*c* = 14.8160 (11) Å
β = 110.846 (6)°
*V* = 922.44 (13) Å^3^
*Z* = 2
Mo *K*α radiation
μ = 0.41 mm^−1^
*T* = 150 K
0.45 × 0.35 × 0.17 mm

#### Data collection

Stoe IPDS II diffractometer
Absorption correction: numerical (*X-RED32*; Stoe & Cie, 2001 ▶) *T*_min_ = 0.837, *T*_max_ = 0.936
11528 measured reflections
2113 independent reflections
1624 reflections with *I* > 2σ(*I*)
*R*_int_ = 0.114

#### Refinement

*R*[*F* ^2^ > 2σ(*F* ^2^)] = 0.039
*wR*(*F* ^2^) = 0.087
*S* = 1.03
2113 reflections
120 parameters
H-atom parameters constrained
Δρ_max_ = 0.38 e Å^−3^
Δρ_min_ = −0.42 e Å^−3^

### 

Data collection: *X-AREA* (Stoe & Cie, 2002 ▶); cell refinement: *X-AREA*; data reduction: *X-AREA*; program(s) used to solve structure: *SHELXS97* (Sheldrick, 2008 ▶); program(s) used to refine structure: *SHELXL97* (Sheldrick, 2008 ▶); molecular graphics: *VESTA* (Momma & Izumi, 2011 ▶); software used to prepare material for publication: *publCIF* (Westrip, 2010 ▶).

## Supplementary Material

Crystal structure: contains datablock(s) global, I. DOI: 10.1107/S1600536811055516/nc2261sup1.cif

Structure factors: contains datablock(s) I. DOI: 10.1107/S1600536811055516/nc2261Isup2.hkl

Additional supplementary materials:  crystallographic information; 3D view; checkCIF report

## Figures and Tables

**Table 1:** Hydrogen-bond geometry (Å, °)

*D*—H⋯*A*	*D*—H	H⋯*A*	*D*⋯*A*	*D*—H⋯*A*
N3—H3⋯O4	0.88	2.12	2.989 (2)	167
N6—H6*A*⋯O1^i^	0.92	1.77	2.6745 (19)	168
N6—H6*B*⋯O2^ii^	0.92	2.13	2.9265 (17)	145
N6—H6*B*⋯O5^ii^	0.92	2.40	3.2141 (19)	147

Symmetry codes: (i) 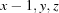; (ii) 
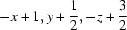
.

**Fig. 1.**
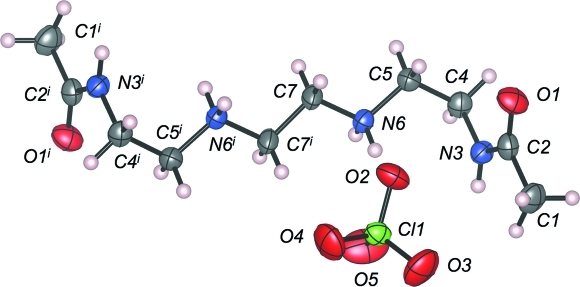
Crystal structure of the title compound with labeling and displacement ellipsoids drawn at the 75% probabiliyt level. [symmetry code: (i) -x, -y - z + 1].

**Fig. 2.**
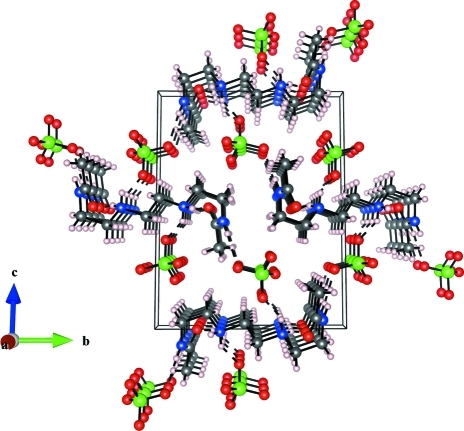
Crystal structure of the title compound with view along the a axis. Hydrogen bonding interactions are shown as dashed lines.

**Fig. 3.**
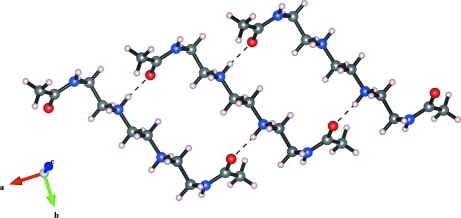
The strands of (H2DAT)2+ cations viewed along the a axis. The dashed bonds indicate the hydrogen bonds.

## supplementary crystallographic information

### Comment

As part of a larger project focusing on TETA and its metabolites as pharmaceutical chelating agents in Diabetes treatment (Cooper *et al.*, 2004, Gong *et al.* 2006, 2008, Jüllig *et al.* 2007, Lu *et al.* 2010), we previously published the detection of a new group of TETA metabolites, *N*^1^-Monoacetyltriethylenetetramine (MAT) and the N^1^, *N*^10^-Diacetyltriethylenetetramine (DAT) (Lu *et al.* 2007), as well as just recently the development of a new selective synthetic route and the characterization of the DAT mono- and dihydrochloride salts (Wichmann *et al.*, 2011).

TETA and its metabolites belong into the polyamine family, ambivalent and multidentate ligands, which are well known for their ability to form a variety of interesting open-chain, macrocyclic and three-dimensional architectures. TETA salts exist in variable protonation states with different anionic species (Ilioudis, *et al.* 2000, 2002, 2003, Elaoud *et al.* 1999, Fu *et al.* 2005, Wichmann *et al.* 2007). Therefore, we investigated the metabolite forms MAT and DAT towards their protonation and complexation behaviour (Wichmann *et al.*, 2011). The obtained crystal structure of the new DAT salt [(H_2_DAT) * 2 ClO_4_] is described in this paper.

The (H_2_DAT)^2+^ cations are arranged as a linear symmetric chain with the terminal NH—CO—CH_3_ groups in *trans*-position to each other (Fig. 1).

The crystal structure consists of a three-dimensional-network, containing alternating assembly of two-dimensional-layers of (H_2_DAT)^2+^ cations (Fig. 2) and the ClO_4_^-^ anions. The (H_2_DAT)^2+^ cations form linear strands along [100] (Fig. 3), connected *via* hydrogen bonding between the acetyl groups and the amine groups of neighbouring (H_2_DAT)^2+^ cations, with a C2=O1 ··· H6A/N6 distance of 1.767 (1) Å. These linear strands of the (H_2_DAT)^2+^ cations form two-dimensional-layers in the (001) plane. However, the two-dimensional-layers of (H_2_DAT)^2+^ cations and the perchlorate anions were stabilized by a network of intermolecular hydrogen bonds between the NH– and NH_2_-groups and the oxygen atoms of the perchlorate anion, with N6—H6B···O2—Cl1—O4···H3—N3 between 2.126 (1) Å and 2.125 (1) Å, (Table 1). The terminal NH-groups of a (H_2_DAT)^2+^ cation binds to an O-atom of a perchlorate anion, which itself bound to an internal NH-group of another (H_2_DAT)^2+^ cation and *vice versa*. Therefore each (H_2_DAT)^2+^ cation is connected to four different (H_2_DAT)^2+^ cations, two from the above and two from the below layer.

### Experimental

The DAT * 2 HCl powder material was synthesized by CarboGen, Switzerland according to literature procedure (Jonas *et al.* 2006, Wichmann *et al.* 2011). Cu(ClO_4_)_2_ is commercially available and was used as received. Crystals of the title compound were grown by slow evaporation of an aqueous solution of DAT * 2 HCl and Cu(ClO_4_)_2_ in stoichiometric ratio in water over a period of 6 weeks.

### Refinement

H atoms bonded to C and N atoms were positioned geometrically (C—H = 0.98–0.99 Å, N—H = 0.88–0.92 Å) and refined using a riding-model approximation, with *U*_iso_(H) = 1.5 *U*_eq_(C, N).

### Crystal data

**Table:**

C_10_H_24_N_4_O_2_^2+^·2ClO_4_^−^	*F*(000) = 452
*M**_r_* = 431.23	*D*_x_ = 1.553 Mg m^−^^3^
Monoclinic, *P*2_1_/*c*	Mo *K*α radiation, λ = 0.71073 Å
Hall symbol: -P 2ybc	Cell parameters from 12054 reflections
*a* = 6.0888 (5) Å	θ = 4.7–59.0°
*b* = 10.9415 (9) Å	µ = 0.41 mm^−^^1^
*c* = 14.8160 (11) Å	*T* = 150 K
β = 110.846 (6)°	Not regular, colourless
*V* = 922.44 (13) Å^3^	0.45 × 0.35 × 0.17 mm
*Z* = 2	

### Data collection

**Table:**

Stoe IPDS II diffractometer	2113 independent reflections
Radiation source: fine-focus sealed tube	1624 reflections with *I* > 2σ(*I*)
graphite	*R*_int_ = 0.114
Image plate detector scans	θ_max_ = 27.5°, θ_min_ = 2.4°
Absorption correction: numerical (*X-RED32*; Stoe & Cie, 2001)	*h* = −7→6
*T*_min_ = 0.837, *T*_max_ = 0.936	*k* = −14→14
11528 measured reflections	*l* = −19→19

### Refinement

**Table:**

Refinement on *F*^2^	Secondary atom site location: difference Fourier map
Least-squares matrix: full	Hydrogen site location: inferred from neighbouring sites
*R*[*F*^2^ > 2σ(*F*^2^)] = 0.039	H-atom parameters constrained
*wR*(*F*^2^) = 0.087	*w* = 1/[σ^2^(*F*_o_^2^) + (0.0435*P*)^2^ + 0.0412*P*] where *P* = (*F*_o_^2^ + 2*F*_c_^2^)/3
*S* = 1.03	(Δ/σ)_max_ = 0.001
2113 reflections	Δρ_max_ = 0.38 e Å^−^^3^
120 parameters	Δρ_min_ = −0.42 e Å^−^^3^
0 restraints	Extinction correction: *SHELXL97* (Sheldrick, 2008), Fc^*^=kFc[1+0.001xFc^2^λ^3^/sin(2θ)]^-1/4^
Primary atom site location: structure-invariant direct methods	Extinction coefficient: 0.0082 (19)

### Special details

**Table:**

Geometry. All e.s.d.'s (except the e.s.d. in the dihedral angle between two l.s. planes) are estimated using the full covariance matrix. The cell e.s.d.'s are taken into account individually in the estimation of e.s.d.'s in distances, angles and torsion angles; correlations between e.s.d.'s in cell parameters are only used when they are defined by crystal symmetry. An approximate (isotropic) treatment of cell e.s.d.'s is used for estimating e.s.d.'s involving l.s. planes.
Refinement. Refinement of *F*^2^ against ALL reflections. The weighted *R*-factor *wR* and goodness of fit *S* are based on *F*^2^, conventional *R*-factors *R* are based on *F*, with *F* set to zero for negative *F*^2^. The threshold expression of *F*^2^ > σ(*F*^2^) is used only for calculating *R*-factors(gt) *etc*. and is not relevant to the choice of reflections for refinement. *R*-factors based on *F*^2^ are statistically about twice as large as those based on *F*, and *R*- factors based on ALL data will be even larger.

### Fractional atomic coordinates and isotropic or equivalent isotropic displacement parameters (Å^2^)

**Table:**

	*x*	*y*	*z*	*U*_iso_*/*U*_eq_	
O1	0.7492 (2)	−0.27758 (14)	0.49858 (8)	0.0292 (3)	
N3	0.4849 (2)	−0.35281 (14)	0.55667 (8)	0.0203 (3)	
H3	0.4552	−0.3783	0.6075	0.030*	
N6	0.1286 (2)	−0.15588 (13)	0.49690 (8)	0.0180 (3)	
H6A	0.0102	−0.2008	0.5058	0.027*	
H6B	0.2493	−0.1497	0.5556	0.027*	
C1	0.8854 (3)	−0.3315 (2)	0.66635 (12)	0.0306 (4)	
H1A	0.9869	−0.4013	0.6669	0.046*	
H1B	0.8082	−0.3455	0.7133	0.046*	
H1C	0.9805	−0.2570	0.6835	0.046*	
C2	0.7025 (3)	−0.31761 (17)	0.56731 (10)	0.0212 (3)	
C4	0.2944 (3)	−0.35009 (17)	0.46258 (10)	0.0218 (4)	
H4A	0.1589	−0.3965	0.4668	0.033*	
H4B	0.3476	−0.3914	0.4145	0.033*	
C5	0.2149 (3)	−0.22157 (17)	0.42777 (10)	0.0211 (3)	
H5A	0.3478	−0.1757	0.4205	0.032*	
H5B	0.0875	−0.2255	0.3637	0.032*	
C7	0.0392 (3)	−0.03159 (17)	0.46280 (11)	0.0230 (4)	
H7A	−0.0947	−0.0376	0.4008	0.035*	
H7B	0.1647	0.0169	0.4518	0.035*	
Cl1	0.43802 (6)	−0.55724 (4)	0.77065 (2)	0.02264 (14)	
O2	0.4296 (2)	−0.55967 (14)	0.86654 (8)	0.0337 (3)	
O3	0.2285 (3)	−0.60503 (17)	0.70295 (10)	0.0478 (4)	
O4	0.4650 (3)	−0.43253 (15)	0.74660 (10)	0.0461 (4)	
O5	0.6382 (3)	−0.62675 (18)	0.77258 (10)	0.0507 (5)	

### Atomic displacement parameters (Å^2^)

**Table:**

	*U*^11^	*U*^22^	*U*^33^	*U*^12^	*U*^13^	*U*^23^
O1	0.0253 (6)	0.0377 (9)	0.0282 (5)	−0.0049 (6)	0.0139 (5)	−0.0020 (5)
N3	0.0192 (6)	0.0211 (8)	0.0200 (6)	0.0012 (6)	0.0062 (5)	0.0024 (5)
N6	0.0164 (6)	0.0183 (7)	0.0180 (5)	0.0012 (6)	0.0044 (4)	0.0009 (5)
C1	0.0222 (8)	0.0346 (12)	0.0290 (8)	0.0027 (8)	0.0020 (6)	0.0014 (8)
C2	0.0204 (7)	0.0180 (9)	0.0251 (7)	0.0018 (7)	0.0081 (6)	−0.0024 (6)
C4	0.0192 (7)	0.0211 (9)	0.0226 (7)	−0.0001 (7)	0.0041 (5)	−0.0031 (6)
C5	0.0214 (7)	0.0231 (9)	0.0185 (6)	0.0032 (7)	0.0069 (5)	0.0001 (6)
C7	0.0251 (8)	0.0201 (9)	0.0265 (7)	0.0083 (7)	0.0124 (6)	0.0060 (6)
Cl1	0.0200 (2)	0.0276 (2)	0.01913 (18)	0.00163 (17)	0.00557 (13)	0.00246 (15)
O2	0.0382 (7)	0.0424 (9)	0.0237 (6)	0.0036 (7)	0.0150 (5)	0.0075 (5)
O3	0.0355 (8)	0.0518 (11)	0.0400 (7)	−0.0072 (8)	−0.0063 (6)	−0.0065 (7)
O4	0.0564 (9)	0.0378 (10)	0.0441 (7)	−0.0072 (8)	0.0180 (7)	0.0166 (7)
O5	0.0381 (8)	0.0661 (13)	0.0463 (8)	0.0256 (8)	0.0130 (6)	−0.0092 (8)

### Geometric parameters (Å, °)

**Table:**

O1—C2	1.231 (2)	C4—C5	1.517 (2)
N3—C2	1.334 (2)	C4—H4A	0.9900
N3—C4	1.4622 (17)	C4—H4B	0.9900
N3—H3	0.8800	C5—H5A	0.9900
N6—C7	1.485 (2)	C5—H5B	0.9900
N6—C5	1.492 (2)	C7—C7^i^	1.515 (3)
N6—H6A	0.9200	C7—H7A	0.9900
N6—H6B	0.9200	C7—H7B	0.9900
C1—C2	1.501 (2)	Cl1—O3	1.4129 (13)
C1—H1A	0.9800	Cl1—O5	1.4284 (15)
C1—H1B	0.9800	Cl1—O4	1.4344 (16)
C1—H1C	0.9800	Cl1—O2	1.4401 (12)
			
C2—N3—C4	121.60 (13)	N3—C4—H4B	109.0
C2—N3—H3	119.2	C5—C4—H4B	109.0
C4—N3—H3	119.2	H4A—C4—H4B	107.8
C7—N6—C5	112.56 (12)	N6—C5—C4	111.17 (13)
C7—N6—H6A	109.1	N6—C5—H5A	109.4
C5—N6—H6A	109.1	C4—C5—H5A	109.4
C7—N6—H6B	109.1	N6—C5—H5B	109.4
C5—N6—H6B	109.1	C4—C5—H5B	109.4
H6A—N6—H6B	107.8	H5A—C5—H5B	108.0
C2—C1—H1A	109.5	N6—C7—C7^i^	110.03 (16)
C2—C1—H1B	109.5	N6—C7—H7A	109.7
H1A—C1—H1B	109.5	C7^i^—C7—H7A	109.7
C2—C1—H1C	109.5	N6—C7—H7B	109.7
H1A—C1—H1C	109.5	C7^i^—C7—H7B	109.7
H1B—C1—H1C	109.5	H7A—C7—H7B	108.2
O1—C2—N3	121.11 (13)	O3—Cl1—O5	111.45 (11)
O1—C2—C1	122.39 (15)	O3—Cl1—O4	109.27 (10)
N3—C2—C1	116.49 (14)	O5—Cl1—O4	109.82 (11)
N3—C4—C5	113.08 (13)	O3—Cl1—O2	110.69 (9)
N3—C4—H4A	109.0	O5—Cl1—O2	107.47 (8)
C5—C4—H4A	109.0	O4—Cl1—O2	108.06 (10)

Symmetry codes: (i) −*x*, −*y*, −*z*+1.

### Hydrogen-bond geometry (Å, °)

**Table:**

*D*—H···*A*	*D*—H	H···*A*	*D*···*A*	*D*—H···*A*
N3—H3···O4	0.88	2.12	2.989 (2)	167
N6—H6A···O1^ii^	0.92	1.77	2.6745 (19)	168
N6—H6B···O2^iii^	0.92	2.13	2.9265 (17)	145
N6—H6B···O5^iii^	0.92	2.40	3.2141 (19)	147

Symmetry codes: (ii) *x*−1, *y*, *z*; (iii) −*x*+1, *y*+1/2, −*z*+3/2.
